# Essential Oils Obtained from Sicilian *Citrus reticulata* Blanco By-Products: Antibacterial and Allelopathic Activity

**DOI:** 10.3390/plants13243527

**Published:** 2024-12-17

**Authors:** Anna Geraci, Alessia Postiglione, Francesco Sgadari, Rosario Schicchi, Natale Badalamenti, Maurizio Bruno, Adriana Basile, Martina Dentato, Viviana Maresca

**Affiliations:** 1 Department of Biological, Chemical and Pharmaceutical Sciences and Technologies (STEBICEF), Università degli Studi di Palermo, Viale delle Scienze, ed. 17, 90128 Palermo, Italy; anna.geraci@unipa.it (A.G.); natale.badalamenti@unipa.it (N.B.); maurizio.bruno@unipa.it (M.B.); 2 Department of Biology, University of Naples Federico II, Complesso University Monte Sant’Angelo, Via Cinthia 4, 80126 Napoli, Italy; alessia.postiglione@unina.it (A.P.); martina.dentato@unina.it (M.D.); 3 Department of Agricultural, Food and Forest Sciences (SAAF), Università degli Studi di Palermo, Viale delle Scienze, ed. 4, 90128 Palermo, Italy; francesco.sgadari@unipa.it (F.S.); rosario.schicchi@unipa.it (R.S.); 4 National Biodiversity Future Center (NBFC), 90133 Palermo, Italy; 5 Department of Life Science, Health, and Health Professions, University of Rome “Link Campus”, 00165 Rome, Italy; v.maresca@unilink.it

**Keywords:** *Citrus reticulata* Blanco, Rutaceae, essential oils, limonene, antibacterial activity, allelopathic effect

## Abstract

Mandarin, one of the winter fruits commonly used in the preparation of foods and juices, is a fruit native to China and Southeast Asia. In this work, essential oils (EOs) obtained from by-products of the *Citrus reticulata* Blanco flavedo of five cultivars present and cultivated within the Botanical Garden of Palermo were chemically and biologically studied: *C. reticulata* ‘Avana’ (**C1**), *C. reticulata* ‘Tardivo di Ciaculli’ (**C2**), *C. reticulata* ‘Bombajensis’ (**C3**), *C. reticulata* ‘Aurantifolia’ (**C4**), and *C. reticulata* ‘Padre Bernardino’ (**C5**). The GC and GC-MS analysis performed on all the extracted samples clearly highlighted the notable presence of limonene, a characteristic hydrocarbon monoterpene of EOs of the *Citrus* genus. **C1**, **C2**, **C3**, **C4**, and **C5** were tested in relation to their possible antibacterial and allelopathic activity, also highlighting the activity of limonene, the main compound. For the antibacterial activity, eight different bacterial strains were used, both Gram-positive and Gram-negative (*Staphylococcus aureus*, *Proteus vulgaris*, *Klebsiella pneumoniae*, *Enterobacter cloacae*, *Pseudomonas aeruginosa*, *Escherichia coli*, *Salmonella typhi*, and *Enterobacter aerogens*). For the allelopathic effect, two model systems were chosen: the germination of radish seeds (*Raphanus sativus* L.) and of spores in the moss *Tortula muralis* (Hedw.). The EOs from all cultivars showed pronounced antibacterial effects against all strains with an MIC comprised in the range of 16–256 μg/mL. Limonene showed the highest activity with an MIC between 4 and 16. The allelopathic effects showed a decrease in the percentage of seed germination, root, and epicotyl growth on *Raphanus* and a strong reduction in the germination of *Tortula* spores with an alteration in the development of the protonema. Limonene showed the same but more intense allelopathic activity.

## 1. Introduction

*Citrus* is one of the most important horticultural crops, with a worldwide production of over 100 million tonnes per year [1]. Their fruits, largely consumed around the world, contain different types of phytochemicals such as carotenoids and flavonoids, and they are an excellent source of vitamin C, which is a powerful natural antioxidant. Moreover, they are a good source of dietary fibres that improve human health, reducing the risk of several chronic degenerative diseases [2,3,4].

Mandarin (*Citrus reticulata* Blanco) is native to China and south-eastern Asia, and it is one of the basic taxa of *Citrus*, together with pummelo [*C. maxima* (Burm.) Merr.], citron (*C. medica* L.), and a wild Papeda species (*C. micrantha* Wester, sin. *Citrus hystrix* DC.) based on biochemical polymorphism, karyotype investigations, and especially on molecular markers and genome sequencing [5,6,7,8]. All cultivated *Citrus* fruits originated from these species. Recent genomic and molecular marker studies revealed that several modern mandarins are not pure *C. reticulata* but are introgressed by *C. maxima* genome fragments [9,10,11].

According to Webber [12], the first appearance of mandarin in Europe dates back to the introduction of two cultivars from Canton (China) to England in 1805.

In Italy, the largest *Citrus* fruit cultivations are in Sicily, where the production is almost 4 million tons per year, mostly oranges, lemons, mandarins, and grapefruits. The *Citrus* crop could be considered, in fact, a major part of the Sicilian economy. In particular, the production of mandarins was about 60,000 t in 2023 [13].

As Lo Piccolo reports [14], the mandarin was introduced to Palermo in 1810 when Louis Philippe d’Orleans sent two plants to Ferdinand of Bourbon. In the following years, it was cultivated at the “Real Tenuta della Favorita”, and in 1817, the botanist Giovanni Gussone introduced ten saplings, purchased in Malta, to the experimental and acclimatisation garden of Boccadifalco. The mandarin arrived at the Botanical Garden of Palermo probably around 1820 and subsequently spread to the countryside around the town and, in particular, to the “Conca d’Oro” area [14].

At a phytochemical level, EOs of the *Citrus* genus are characterised by the high presence of limonene, a hydrocarbon monoterpene, and by small quantities of various compounds such as *β*-myrcene, linalool, and *α*-terpineol [15].

The particular abundance of this compound has allowed several studies to be carried out both in vitro and in vivo. In fact, the biological properties of the *Citrus* EOs obtained from different parts of the plant, such as fruits, seeds, leaves, and flowers, as well as by-products such as flavedo (coloured outer layer) and albedo (inner layer), have been investigated. Activities such as antioxidant [16,17], antidiabetic [18], antimicrobial [19], anti-obesity [20], anti-inflammatory [21], cytotoxic [22], and anxiolytic [23] have been extensively investigated. The particular richness of healthy, natural compounds allows the use of this species for the treatment of various pathologies related to the heart, liver, and intestinal system, and against obesity [21,22,23,24].

The by-products, primarily the peels obtained from the agri-food industry that processes *Citrus* fruits, have an important socio-economic interest. Several studies, in fact, have shown that from the recycling of this waste, biologically active compounds such as flavones, flavonols, chalcones, terpenes, vitamins, and limonoids can be obtained [16,25,26].

Finally, the large-scale use of EOs is noteworthy, both for the production of perfumes (the cosmetic field) and for use in the healthcare and culinary fields [27]. In fact, *Citrus* species have an important economic–commercial value. These fruits are mainly consumed fresh, but they are also used for the production of drinks and fruit juices. The by-products, i.e., the dehydrated pulp, peels, leaves, and aerial parts, are instead used for their bioactive potential to obtain products that can be used for animal feed or even for healthcare [28]. Among the main producing countries are Italy, especially in Sicily, some member states of the USA, and several countries in Southeast Asia. EOs obtained mainly from the flavedo of these citrus fruits are widely used as flavourings and accepted by the Food and Drug Administration in the form of additives for food products, especially thanks to the excellent antimicrobial properties they possess [29]. Finally, there is a very important industrial application with the use of EOs in the formulation of hygiene products as alternatives to the use of traditional solvents in the production of cosmetics and in perfumes [30].

Among the many activities studied so far for the essential oils of *Citrus reticulata* Blanco, allelopathic activity does not appear, which could represent an interesting possibility for using the waste products of fruit processing.

**Figure 1:**
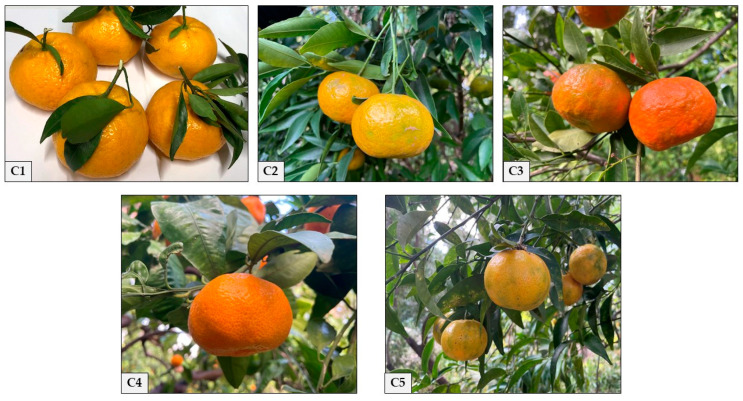
The five *Citrus reticulata* Blanco cultivars collected in the Palermo Botanical Garden: *C. reticulata* ‘Avana’ (**C1**), *C. reticulata* ‘Tardivo di Ciaculli’ (**C2**), *C. reticulata* ‘Bombajensis’ (**C3**), *C. reticulata* ‘Aurantifolia’ (**C4**), and *C. reticulata* ‘Padre Bernardino’ (**C5**).

In this context, the EOs of five Sicilian cultivars grown within the Palermo Botanical Garden, including *C. reticulata* ‘Avana’ (**C1**), *C. reticulata* ‘Tardivo di Ciaculli’ (**C2**), *C. reticulata* ‘Bombajensis’ (**C3**), *C. reticulata* ‘Aurantifolia’ (**C4**), and *C. reticulata* ‘Padre Bernardino’ (**C5**) (Figure 1), were chemically analysed by GC and GC-MS analyses, and were tested for their antibacterial and allelopathic activity.

In relation to the first, eight strains were used, both Gram-positive and Gram-negative (*Staphylococcus aureus*, *Proteus vulgaris*, *Klebsiella pneumoniae*, *Enterobacter cloacae*, *Pseudomonas aeruginosa*, *Escherichia coli*, *Salmonella typhi*, and *Enterobacter aerogens*). For the second, two model systems, the germination of radish seeds (*Raphanus sativus* L.) and of spores in the moss *Tortula muralis* (Hedw.), were chosen. In addition to the inhibitory activity on germination in both systems, the effect on morphology was evaluated with respect to the development of radish seedlings in *R. sativus,* as root and epicotyl growth, and as an extension of the hairy area, and to the alteration in the development of the protonema and brood and tmema cells in *T. muralis*, specific resistance cells produced by the protonema of mosses in response to various stressors.

## 2. Results and Discussion

### 2.1. Chemical Composition of EOs (C1–C5)

The hydrodistillates (EOs) obtained from the flavedos of the different Sicilian cultivars of *C. reticulata* (**C1**–**C5**) were all yellow oils. In total, thirty-six metabolites were identified by GC and GC-MS analysis and tabulated in Table 1 based on retention times, linear retention indices on the apolar DB-5MS column, and clearly clustered into different chemical classes, including monoterpene hydrocarbons, oxygenated monoterpenes, sesquiterpene hydrocarbons, oxygenated sesquiterpenes, and other compounds.

An examination of the results (Table 1) revealed how the five different EOs (**C1**–**C5**) were characterised by the massive presence of hydrocarbon monoterpene compounds with a percentage range varying from 96.69% to 98.78%. Figure S1 shows a histogram highlighting the chemical differences of samples **C1**–**C5**. All the different samples (**C1**–**C5**) are characterised by the abundance of limonene (70.74–86.80%), with a variable that may have depended on the vegetative season and the ripening of the harvested fruits [31].

*α*-Pinene, *β*-pinene, *β*-myrcene, *γ*-terpinene, and terpinolene were other metabolites present in all the EOs examined and also contributed moderately to the percentage increase in the class of hydrocarbon monoterpenes. Moderate amounts of oxygenated monoterpenes (0.37–1.04%) were detected in all samples, while only sample **C2** presented oxygenated sesquiterpene compounds (0.25%) and other metabolites (others class) (0.12%).

From research conducted on the major scientific information systems such as Scopus, SciFinder, and Google Scholar, the presence of chemical information and the biological applicability of the EOs for the varieties ‘Avana’, ‘Tardivo di Ciaculli’, ‘Bombajensis’, ‘Aurantifolia’, and ‘Padre Bernardino’ was not evident. However, there are several works that have reported the chemical composition of EOs obtained from waste, such as *C. reticulata* flavedo. By evaluating the chemical composition of *C. reticulata* EOs [32], a high percentage of limonene (60.74%) and the moderate presence of monoterpene compounds, such as terpinene and myrcene, were found. High amounts of limonene have also been found in recent studies on EOs obtained from *C. reticulata* samples collected and cultivated in China [33], India [34], and Brazil [35], underlining that there is no geographic difference resulting in true chemical variation.

### 2.2. Antibacterial Activity

**C1**–**C5** showed inhibition activity against all the bacterial strains tested (MIC between 64 and 256 μg/mL). *Staphylococcus aureus* was the most sensitive bacterium (MIC between 16 and 32 μg/mL), followed by *Proteus vulgaris* (MIC between 32 and 128 μg/mL), while *Pseudomonas aeruginosa* and *Salmonella typhi* were the least sensitive (MIC between 128 and 256 μg/mL). EOs with an MIC of 256 μg/mL were inactive. (Table 2).

All EOs showed inhibitory activity against all bacterial strains tested, especially with *S. aureus,* the most sensitive bacterium, while *Salmonella* was the least sensitive. Among the numerous compounds present in EOs, it was decided to test for limonene because it is present in considerable quantities and because its antibacterial activity and its ability to interact with membranes, a possible target for an allelopathic activity, are known [36,37]. Furthermore, since it has been demonstrated that limonene acts in *S. aureus* on numerous essential cellular activities (destruction of the cellular morphology and integrity of the cell wall), loss of biological macromolecules (nucleic acids and proteins, damage to the cell membrane with increased permeability and reduction of its potential, reduction of respiratory metabolic activity, and a slowing down of metabolism with metabolic dysfunction) [37], it is possible that all these effects may be responsible for the antibacterial activity demonstrated to us both for EOs and for limonene. Furthermore, these same effects, if demonstrated in plant cells, could give an idea of the possible mechanism of action of the allelopathic effect shown by us. The sometimes-close value of pure limonene and EOs, in which limonene represents only a good percentage, may be due to the effect of the other oils present and their possible synergistic effect. Finally, it is intriguing to consider that in **C3** and **C4,** the percentage of limonene is the highest.

### 2.3. Allelopathic Activity

#### 2.3.1. Raphanus Sativus Seed Germination

**Figure 2:**
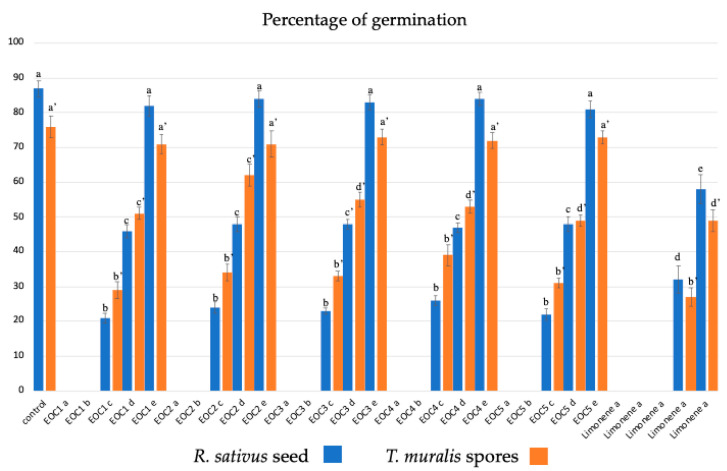
Effect of **C1**–**C5** and limonene on the percentage of germination of the *Raphnus sativus* seed and *Tortula muralis* spores a = 10 µg/mL; b = 1 µg/mL; c = 0.1 µg/mL; d = 0.01 µg/mL; e = 0.001 µg/mL. Data were presented as mean and standard error, and they were analysed with a paired *t*-test. Bars not accompanied by the same letter were significantly different at *p* < 0.05.

Regarding the germination of *R. sativus* seeds, EOs at 10 and 1 μg/mL completely inhibited seed germination, while the other concentrations inhibited the percentage of seed germination, the length of the hypocotyl-root axis, and hair growth in a dose-dependent manner (Figure 2 and Table 3). In the treated samples, the most evident morphological alteration was the thickening of the root apex. Furthermore, the length of the hair zone and hair growth were reduced, corresponding to early developmental stages (e.g., a 2-day-old root was comparable to a 12-h-old root).

**Figure 3:**
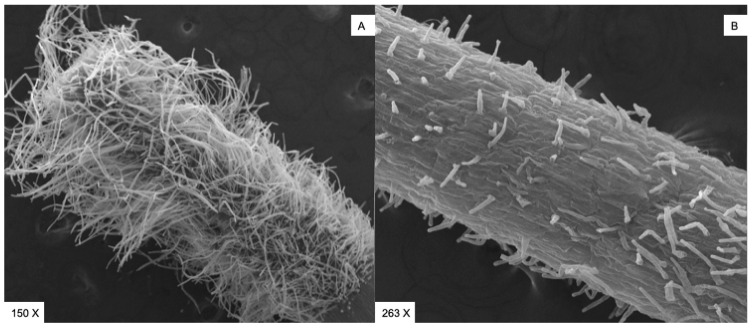
(**A**) SEM micrograph of the control samples after 7 days of culture showing a large hairy area densely populated by root hairs. (**B**) SEM micrograph of the samples treated with **C1** at 0.1 µg/mL after 7 days of culture showing only a few short hairs.

As can be seen from studies carried out using SEM (Scanning Electron Microscopy, Cambridge 250 Mark 3) in the control sample (A), an extensive hairy area is evident, while in the sample treated with **C1** (0.1 mM) (B), only a few short hairs appear to form on the rhizoderm (Figure 3).

The potential impact of different EOs of *C. genuson* and limonene on plant growth may occur through several mechanisms. They may inhibit seed germination by disrupting cell membranes or altering the hormonal balance required for germination, or, even at non-toxic concentrations, they may suppress root elongation and shoot development, thereby reducing overall plant growth.

High concentrations of limonene may cause a significant decrease in the percentage of germinated seeds. This may be due to the potential toxicity of the chemical to the embryo or its interference with biochemical processes required for germination. Even at lower concentrations, limonene may delay the time it takes for seeds to germinate, thereby inhibiting early growth.

#### 2.3.2. Moss Spore Germination

Under control conditions, the moss *T. muralis* began to germinate already after 3 days and reached its maximum percentage of germination (80–90%) after 14 days. The germination pattern, unipolar or bipolar, showed chloronemata (with transverse septa and numerous chloroplasts) as the first type of filament originating from the spore. After 21 days, chloronemata and caulonemata (with oblique septa, a few plastids arranged in longitudinal rows, and an apical exclusion zone) were evident, while brood and tmema cells appeared after 30 days. **C1**–**C5** at 10 and 1 µg/mL inhibited spore germination completely (Figure 2 and Table 3). The other concentrations inhibited both germination percentage and protonemata development in a dose-dependent manner, showing the highest inhibition values for **C1** and **C5**. In the moss, the main morphological changes were swollen and shorter cells and early development of brood and tmema cells after 14 days in culture, with higher concentrations of both cell types for **C1**- and **C5**-treated samples. Limonene showed the same but more intense activity.

Finally, also with regard to allelopathic activity, **C1** and **C5** showed the highest inhibition values both for seed germination and plant development in *Raphanus* and for spore germination and protonema development in *Tortula*.

Brood cells are resistant structures due to their thick walls, abundant nutrient reserves, and a low surface/volume ratio that reduces exchanges [38,39,40]. They are produced in response to flavonoids and to heavy metal (lead) stress [41]. The frequent occurrence of these diaspores after exposure to toxic substances confirms that they are a common stress response of moss protonemata. Also, in this case, the dose-dependent production of brood cells in response to treatment with essential oils could indicate an increase in the resistance of the moss model, thanks to the production of these forms of resistance.

Abscission, or tmema, cells are specialised cells causing protonemata breakage and liberation of short filaments [40,41,42,43]. Our findings regarding both tmema cell formation and toxic effects after treatment with *C. reticulata* Blanco essential oil are consistent with the literature data regarding these cells as a means of propagation of mosses in adverse conditions, such as scant nourishment, low Caþ2 concentration, and ageing and heavy metals [41,44]. Just like the brood cells, the tmema cells, which we highlighted as a dose-dependent increase after treatment with EOs, can be considered a defence and resistance mechanism of the moss protonema.

The allelopathic activity of *Citrus reticulata* essential oils has been shown in previous works and has been demonstrated against seed germination and seedling growth of *Heliantus annus* (sunflower), *Portulaca oleracea* (purslane), *Lupinus albus* (field lupine), and *Malva parviflora* (Egyptian mallow), and showing complete inhibition of seed germination and seedling growth of *H. annus* and *M*. *parviflora* at all concentrations [45].

Furthermore, it has been reported that EOs from flowering aerial parts of *Dracocephalum kotschyi* that showed a high amount of EOs with a significant percentage of limonene-10-al and limonene exerts inhibitory effects on seed germination and seedling growth of *Amaranthus retroflexus* L. and *Chenopodium album* L. [46].

Finally, *Xanthium sibiricum* EOs and two of its constituents (including limonene) showed allelopathic activity on the germination of *Amaranthus retroflexus* L. and *Poa annua* L., demonstrating a strong inhibitory activity on seedling growth and root elongation in both species [47].

The contemporary occurrence of different bioactivities (allelopathic and antibacterial) is shared by other plant-derived substances [48,49]. *S. italica* EOs and flavonoids from *Castanea sativa*, for example, have been observed to inhibit both bacterial and *R. sativus* root growth, and flavonoids from mosses inhibited spore germination and protonemal growth in *T. muralis* and seed germination and epicotyl growth in *R. sativus* [50].

Like seeds, spores may show reduced germination under the influence of different EOs of *C. genuson* and limonene, especially if the compound directly interferes with physiological processes involved in spore activation. Furthermore, it may also affect the growth rate of spores once they begin to germinate, potentially reducing the establishment of new plants. Limonene inhibits germination and growth, which would suggest that citrus plants may use allelopathy as a competitive mechanism, potentially influencing plant community dynamics in their natural habitats.

## 3. Materials and Methods

### 3.1. Plant Materials

The five cultivars of *Citrus reticulata* analysed, grown in the Botanical Garden of Palermo (38°06′48.39″ N; 13°22′21.68″ E), Sicily (Italy), were harvested between December 2022 and February 2023. In the present work, fruits belonging to the following five cultivars were examined: *C. reticulata* ‘Avana’ (**C1**), *C. reticulata* ‘Tardivo di Ciaculli’ (**C2**), *C. reticulata* ‘Bombajensis’ (**C3**), *C. reticulata* ‘Aurantifolia’ (**C4**), and *C. reticulata* ‘Padre Bernardino’ (**C5**). The samples, identified by Prof. Rosario Schicchi and Prof. Anna Geraci, were stored in the Herbarium Mediterraneum of the Botanical Garden at the University of Palermo (PAL). The voucher number is reported for each cultivar.

*C. reticulata* ‘Avana’ (**C1**) (Voucher No. 109766) is characterised by medium-sized, slightly flattened, globular fruits with a smooth, thin epicarp and a sweet, juicy endocarp (pulp) consisting of easily divisible, orange-coloured segments with few seeds. The leaves are ovate, with a short petiole. It ripens between mid-November and January.

*C. reticulata* ‘Tardivo di Ciaculli’ (**C2**) (Voucher No. 109767) is characterised by a slightly smaller fruit than the ‘Avana’ cv. from which it is derived by bud mutation. It takes its name from Ciaculli, a locality situated in Palermo’s famous Conca d’Oro. The fruit is globular in shape, slightly flattened, and has a smooth, thin, orange epicarp. The flesh is juicy and very sweet, consisting of easily divisible segments with few seeds. It ripens between mid-February and March.

*C. reticulata* ‘Bombajensis’ (**C3**) (Voucher No. 109768) is characterised by medium-sized fruits, slightly flattened and depressed in the distal part, with an irregular, moderately thick, reddish-orange epicarp. The mesocarp is well represented, and the flesh is sweet and juicy, consisting of divisible segments with few seeds. The leaves are ovate-oblong, with a slightly winged petiole. It ripens between mid-January and February.

*C. reticulata* ‘Aurantifolius’ (**C4**) (Voucher No. 109770) is characterised by medium-sized fruit, tending to be globular in shape, with a slightly wrinkled epicarp, a deep orange colour and sweet flesh consisting of easily divisible segments. The leaves are ovate-oblong, wider than in previous cultivars and are similar in shape, width, and length to those of the orange. It ripens between December and January.

*C. reticulata* ‘Padre Bernardino’ (**C5**) (Voucher No. 109769) is characterised by medium-small, slightly flattened fruits with a smooth, thin epicarp and a slightly orange colour. The juicy flesh is slightly sour and consists of easily divisible segments with several seeds. The leaves are elliptical, with a short petiole. It ripens between January and February.

### 3.2. Extraction of EOs

EOs extraction was performed following a reported method [51]. The orange part (flavedo), without the albedo, was obtained using an electric peeler (093209-006-BLCK, 770 Boulevard Guimond, Longueuil Quebec, Canada), was weighed, and subjected to hydro-distillation using Clevenger’s apparatus [52]. Amounts used were 300 g, 212 g, 260 g, 299 g, and 173 g for **C1**, **C2**, **C3**, **C4**, and **C5**, respectively. The oils’ yields were 0.69%, 1.01%, 0.89%, 0.58%, and 0.66% (*v*/*w*) for **C1**, **C2**, **C3**, **C4**, and **C5**, respectively. The obtained EOs were dried with Na_2_SO_4_, stored in appropriate vials, and placed in the freezer at −20 °C until the time of analysis.

### 3.3. GC and GC-MS Analyses

Analysis of EOs was carried out according to the procedure already reported [53]. GC-MS analysis was performed using a Shimadzu QP 2010 plus equipped with an AOC-20i autoinjector (Shimadzu, Kyoto, Japan) gas chromatograph equipped with a FID, a capillary column (DB-5MS) 30 m × 0.25 mm i.d., a film thickness 0.25 μm, and a data processor. The oven program was as follows: the temperature was held at 40 °C for 5 min, then increased at a rate of 2 °C/min up to 260 °C, then isothermal for 20 min. Helium was used as carrier gas (1 mL min^−1^). The injector and detector temperatures were set at 250 and 290 °C, respectively. One μL of EO solution (3% EO/hexane *v*/*v*) was injected in split mode 1:50; MS range 40–600. The settings were as follows: ionisation voltage, 70 eV; electron multiplier energy, 2000 V; transfer line temperature, 295 °C; and solvent delay, 3 min.

Linear retention indices (LRIs) were calculated on DB-5MS retention indices using a mixture of pure *n*-alkanes (C_8_–C_40_), and all the peaks’ compounds were identified by comparison with MS and by comparison to their relative retention indices with WILEY275, NIST 17, ADAMS, and FFNSC2 libraries. The analyses were performed in triplicate, and the results are expressed as the average of three measurements ± standard deviation.

### 3.4. Antimicrobial Assay

The following eight bacterial strains were obtained from the American Type Culture Collection (ATCC; Rockville, MD, USA): *Staphylococcus aureus* (ATCC 13709), (Gram-positive), and *Proteus vulgaris* (ATCC 12454), *Klebsiella pneumoniae* (ATCC 10031), *Enterobacter cloacae* (ATCC 10699), *Pseudomonas aeruginosa* (ATCC 27853), *Escherichia coli* (ATCC 11229), *Salmonella typhi* (ATCC 10699), and *Enterobacter aerogens* (ATCC 13048) (all Gram-negative). Bacterial strains were grown on MH agar plates (DIFCO, Detroit, MI, USA) and suspended in MH broth (DIFCO). The minimum inhibitory concentration (MIC) values were determined using the MH broth-dilution method [54]. The inoculum suspensions were prepared from 6-h broth cultures and adjusted to a 0.5 McFarland standard turbidity. The extracts, sterilised by 0.45-μm Millipore filters, were added to the MH broth medium. Serial 10-fold dilutions were made that furnished a concentration range from 0.01 to 1000 μg/mL for the plant extract. The lowest concentrations of the extract with activity underwent two-fold dilutions for a more accurate measurement of the MIC. The bacterial suspensions were aerobically incubated for 24 h at 37 °C. The MIC was defined as the lowest concentration able to inhibit any visible bacterial growth. Control cultures containing only sterile physiological Tris buffer were also prepared. In addition, MIC values for tetracycline hydrochloride (Pharmacia, Milano, Italy), benzylpenicillin sodium (Cynamid, Catania, Italy) and cefotaxime sodium (Roussel Pharmacia, Milano, Italy) were also determined.

### 3.5. Allelopathic Test

Tests on the allelopathic effects of EOs of the *C. genuson* and limonene on the germination percentage of *R. sativus seeds* (2 days after sowing) and of *T. muralis* spores (14 days after sowing) were conducted.

All tested plants were grown on a modified Mohr medium, as reported in Basile et al. [49], used as a control, and on the same medium with the addition of serial 10-fold dilutions of essential oils of the *Citrus genuson* and limonene to obtain the concentration range (from 0.001 to 10 µg/mL). EOs from the flowerheads of *Sideritis italica* (Miller) Greuter et Burdet, a widespread Mediterranean Lamiaceae, was used as a positive control (Figure S2) because it demonstrated efficacy in inhibiting germination of the plants chosen as “models”, as *R. sativus* and *T. muralis* had already been confirmed in previous experiments [46].

The media were sterilised by filtration through Millipore filters (0.45 mm). The culture solutions were replaced every 2 days. The cultures were kept in a growth chamber with a temperature ranging from 13 °C at night to 20 °C by day, 70% constant relative humidity, and a 16-h light (45 mE m^−2^ s^−1^)/8 h dark photoperiod. The plants were maintained in the growth chamber for 30 days. The experiments were carried out in triplicate.

### 3.6. Seed Germination Tests

Seeds of *R. sativus* L. were surface-sterilised [49] and subsequently washed (10 min) with sterile distilled water. Then, they were put in Petri dishes (5 cm diameter), with 20 seeds per dish, on fine granular washed quartz (Merck, Germany) (10 g), with 10 mL of sterile culture medium.

### 3.7. Moss Spore Germination Test

Mature capsules of *T. muralis* (Hedw.) were surface-sterilised in 70% ethanol (2 min) and 2% NaClO with the addition of a few drops of Triton X-100 (Sigma-Aldrich, USA) (5 min). Subsequently, they were washed (10 min) with sterile distilled water, and the contents of 10 capsules were suspended in 10 mL of sterile distilled water. Aliquots (200 mL) of spore suspension were inoculated in Petri dishes (5 cm diameter) with 10 mL of sterile liquid medium to test **C1**–**C5**.

### 3.8. Evaluation of Oil Effects

Observations were made, and photographs were taken using a Leitz Aristoplan microscope (Leica, Wetzlar, Germany) equipped with differential interference contrast optics (Nomarski) and a Wild Heerbrugg M3Z binocular. Gemmalings were measured using a calibrated, square graticule eyepiece. *R. sativus* seed germination percentages were evaluated by examining 20 seeds per replicate at 2 days, and hypocotyl-root length was measured on 15 roots per replicate at 3 days after inoculation. As for the mosses, *T. muralis*, we monitored spore germination percentage, cell number in the main filament, length of newly formed filaments, type of filaments, order of branch formation, and presence of brood and tmema cells (see below). The moss was visually evaluated every 2 days.

### 3.9. Scanning Electron Microscopy

For scanning electron microscopy (SEM), small pieces of control and EO1 0.1 mM exposed samples were cut from the hairy area using a sharp razor blade. The samples were fixed in 3% glutaraldehyde in a phosphate buffer (65 mM, pH 7.2–7.4) for 2 h at room temperature, post-fixed with 1% osmium tetroxide in the same phosphate buffer for 1.5 h at room temperature and dehydrated with ethanol and critical point drying. The samples were then mounted on aluminium stubs, coated with a thin gold film using an Edward E306 Evaporator, and observed with an FEI (Hillsboro, OR, USA) Quanta 200 ESEM (FEI Company, Hillsboro, OR) in high vacuum mode at 30 kV voltage.

### 3.10. Statistical Analysis

The data are mean values of the three experiments. A one-way ANOVA test was performed. The significance of differences between means was checked by Student’s *t*-test (*p* < 0.05).

## 4. Conclusions

The EOs obtained from flavedo waste of the *Citrus reticulata* Blanco cultivars (**C1**–**C5**) were investigated both chemically and biologically. The analyses carried out by GC-MS highlighted the class of hydrocarbon monoterpenes as the main class, with quantities of limonene varying from 70.83 to 86.73%. Small amounts of oxygenated monoterpenes were found in all samples, while **C2** was the most chemically diverse sample. All EOs, together with the limonene standard, were tested for antibacterial and allelopathic activity and showed pronounced antibacterial effects against all strains with an MIC in the range of 16–256 µg/mL, a strong reduction in the germination of *Tortula spores* with an alteration in the development of the protonema, and a decrease in the percentage of seed germination, root, and epicotyl growth in *Raphanus*. Fairly modest, if not minimal, quantities of sesquiterpene compounds were detected in all samples except for **C3**. EOs of *Citrus reticulata* Blanco can perform a multifaceted defensive action against a wide range of competitors and/or stressors and could be used as a natural base for formulations in the pharmaceutical and/or agricultural fields, avoiding or reducing the use of synthetic compounds.

## Figures and Tables

**Table 1:** Chemical composition flavedo EOs of the five Sicilian cultivars of *C. reticulata* collected in the Palermo Botanical Garden.

No.	Compounds ^a^	LRI ^b^	LRI ^c^	Area (%) ^d^				
				C1	C2	C3	C4	C5
1	Hexanal	803	798	-	-	*-*	*t*	*-*
2	3-Hexen-1-ol	861	857	-	-	*t*	*t*	*-*
3	Nonane	902	900	-	-	*-*	*t*	*-*
4	*α*-Pinene	936	938	3.12 ± 0.13	3.35 ± 0.15	1.46 ± 0.04	1.65 ± 0.05	3.69 ± 0.11
5	*β*-Pinene	978	980	1.83 ± 0.06	1.71 ± 0.05	0.79 ± 0.02	0.81 ± 0.03	2.02 ± 0.08
6	*β*-Myrcene	1003	1005	2.21 ± 0.08	2.09 ± 0.07	2.57 ± 0.08	2.62 ± 0.07	2.08 ± 0.06
7	Limonene	1067	1063	72.89 ± 3.06	70.74 ± 2.91	86.80 ± 3.38	82.22 ± 3.19	71.32 ± 2.94
8	*γ*-Terpinene	1083	1081	17.75 ± 0.57	18.26 ± 0.58	5.87 ± 0.24	8.99 ± 0.29	17.80 ± 0.64
9	Terpinolene	1095	1089	0.98 ± 0.04	0.89 ± 0.04	0.29 ± 0.01	0.40 ± 0.02	1.00 ± 0.03
10	*β*-Linalool	1109	1103	*t*	0.23 ± 0.00	1.11 ± 0.04	0.38 ± 0.01	*t*
11	Nonanal	1112	1008	*-*	*t*	*t*	*t*	*-*
12	Camphor	1147	1145	*-*	*-*	*t*	*-*	*t*
13	*β*-Citronellal	1158	1154	*-*	*t*	*t*	*t*	*t*
14	Terpinen-4-ol	1181	1183	0.22 ± 0.01	0.20 ± 0.00	*t*	*t*	0.25 ± 0.01
15	*p*-Cymene-8-ol	1188	1189	-	-	*-*	*t*	-
16	*α*-Terpinol	1196	1192	0.38 ± 0.01	0.43 ± 0.02	*t*	*t*	0.52 ± 0.02
17	Decanal	1209	1210	*t*	0.11 ± 0.00	*t*	*t*	*t*
18	Octyl acetate	1216	1215	*-*	*-*	*-*	*t*	*-*
19	Thymol methyl ether	1231	1228	*-*	*-*	*t*	*-*	*-*
20	2-Decenal	1265	1267	*-*	*-*	*t*	*-*	*-*
21	Perillal	1272	1271	*-*	*t*	*t*	*t*	*-*
22	1-Decanol	1277	1275	*-*	*-*	*-*	*-*	*t*
23	*p*-Mentha-1(7),8(10)-dien-9-ol	1289	1287	*-*	*t*	*-*	*-*	*-*
24	Thymol	1293	1292	*t*	*t*	*t*	*-*	*t*
25	Undecanal	1308	1306	*-*	*-*	*t*	*t*	*t*
26	*δ*-Elemene	1334	1337	*-*	*-*	*t*	*t*	*-*
27	Citronellol acetate	1352	1354	*-*	*-*	*-*	*t*	*-*
28	Neryl acetate	1361	1363	*-*	*-*	*t*	*t*	*-*
29	*α*-Copaene	1368	1370	*t*	*-*	*t*	*t*	*t*
30	*β*-Elemene	1383	1381	0.20 ± 0.00	0.74 ± 0.03	*t*	0.35 ± 0.01	0.28 ± 0.01
31	*β*-Caryophyllene	1417	1420	*t*	*t*	*-*	*t*	*t*
32	*α*-Caryophyllene	1445	1452	*t*	*-*	*t*	*t*	*-*
33	*α*-Selinene	1485	1483	*t*	*t*	*-*	0.21 ± 0.00	-
34	(*Z,E*)-*α*-Farnesene	1502	1497	*t*	*t*	*t*	0.54 ± 0.02	*t*
35	*δ*-Cadinene	1510	1508	*t*	*-*	*t*	*t*	*t*
36	α-Sinensal	1740	1745	-	0.22 ± 0.00	-	-	*t*
	**Monoterpene Hydrocarbons**			**98.78 ± 3.94**	**97.04 ± 3.80**	**97.78 ± 3.79**	**96.69 ± 3.65**	**97.91 ± 3.86**
	**Oxygenated Monoterpenes**			**0.60 ± 0.02**	**0.74 ± 0.02**	**1.11 ± 0.04**	**0.38 ± 0.01**	**0.77 ± 0.03**
	**Sesquiterpene Hydrocarbons**			**0.20 ± 0.00**	**0.74 ± 0.03**	**-**	**1.10 ± 0.03**	**0.28 ± 0.01**
	**Oxygenated Sesquiterpenes**			**-**	**0.22 ± 0.00**	**-**	**-**	**-**
	**Others**			**-**	**0.11 ± 0.00**	**-**	**-**	**-**
	**Total**			**99.58 ± 3.96**	**98.85 ± 3.85**	**98.89 ± 3.83**	**98.17 ± 3.69**	**98.96 ± 3.90**

^a^ The compounds are classified in order of the linear retention index of the non-polar column DB-5MS. ^b^ LRI calculated for the DB-5MS non-polar column; ^c^ Linear retention indices based on the literature (https://webbook.nist.gov/; accessed on 15 September 2024); ^d^ content is the peak volume percentage of the compounds in the essential oil sample; *t*: traces (<0.05).

**Table 2:** Antibacterial activity (MIC values are expressed as μg/mL) of **C1**–**C5** against several bacterial strains.

	C1	C2	C3	C4	C5	Lim	Ce	Pe	Te
*Staphylococcus aureus*	16	32	32	32	16	4	R	2	0.03
*Proteus vulgaris*	32	128	128	128	32	8	R	16	2
*Klebsiella pneumoniae*	128	128	256	256	128	8	R	0.1	R
*Enterobacter cloacae*	64	128	128	128	64	16	R	R	R
*Escherichia coli*	64	64	128	128	128	8	R	0.1	64
*Pseudomonas aeruginosa*	128	256	128	256	128	4	R	R	16
*Salmonella typhi*	128	256	256	256	128	4	0.5	1	4
*Enterobacter aerogens*	64	128	128	128	64	16	R	R	R

R: no inhibition even at the highest tested concentration; Lim: limonene; Ce: cefotaxime sodium, reference antibiotic; Te: tetracycline hydrochloride; Pe: benzylpenicillin sodium.

**Table 3:** Effect of **C1**–**C5** and limonene on the *R. sativus* hypocotyl-root axis length (cm) and on the cell number of main protonemata filaments, number of brood cells, and number of tmema cells of the *T. muralis*. Data were presented as mean and standard error, and they were analysed with a paired *t*-test. Bars not accompanied by the same letter were significantly different at *p *< 0.05.

Samples	*R. sativus*		*T. muralis*		
	Hypocotyl-Root Axis Length in cm	Length of the Hair Zone	Cell Number of Main Protonemata Filaments	Number of Brood Cells	Number of Tmema Cells
control	2 ± 0.1 a	0.5 ± 0.1 a’	25 ± 2.3 a”	0	0
**C1** ^a^	0	0	0	0	0
**C1** ^b^	0	0	0	0	0
**C1** ^c^	0.3 ± 0.1 b	0	7 ± 1.3 b”	1 ± 0.2 a’’’	0
**C1** ^d^	0.8 ± 0.4 c	0	15 ± 2.6 c”	3 ± 0.9 b’’’	1 ± 0.1 a’’’’
**C1** ^e^	1.7 ± 0.3 a	0.3 ± 0.1 b’	22 ± 3.1 a”	6 ± 1.1 c’’’	3 ± 0.2 b’’’’
**C2** ^a^	0	0	0	0	0
**C2** ^b^	0	0	0	0	0
**C2** ^c^	0.5 ± 0.1 b	0	9 ± 1.8 b”	1 ± 0.1 a’’’	0
**C2** ^d^	1.4 ± 0.4 c	0.2 ± 0.1 b’	18 ± 2.2 c”	2 ± 0.3 b’’’	1 ± 0.1 a’’’’
**C2** ^e^	1.9 ± 0.3 a	0.3 ± 0.1 b’	24 ± 3.3 a”	5 ± 0.4 c’’’	2 ± 0.3 c’’’’
**C3** ^a^	0	0	0	0	0
**C3** ^b^	0	0	0	0	0
**C3** ^c^	0.5 ± 0.1 b	0	8 ± 1.2 b”	1 ± 0.2 a’’’	0
**C3** ^d^	1.4 ± 0.4 c	0.2 ± 0.1 b’	17 ± 2.3 c”	3 ± 0.4 b’’’	1 ± 0.2 a’’’’
**C3** ^e^	1.8 ± 0.3 a	0.3 ± 0.1 b’	24 ± 2.7 a”	4 ± 0.3 c’’’	2 ± 0.3 c’’’’
**C4** ^a^	0	0	0	0	0
**C4** ^b^	0	0	0	0	0
**C4** ^c^	0.5 ± 0.1 b	0	9 ± 2.1 b”	1 ±0.2 a’’’	0
**C4** ^d^	1.5 ± 0.4 c	0.1 ± 0.1 b’	16 ± 2.3 c”	3 ± 0.5 b’’’	1 ± 0.4 a’’’’
**C4** ^e^	1.9 ± 0.3 a	0.2 ± 0.1 b’	24 ± 2.5 a”	4 ± 1.2 c’’’	2 ± 0.6 c’’’’
**C5** ^a^	0	0	0	0	0
**C5** ^b^	0	0	0	0	0
**C5** ^c^	0.4 ± 0.2 b	0	6 ± 1.1 d”	1 ± 0.3 a’’’	0
**C5** ^d^	0.9 ± 0.3 c	0	13 ± 2.8 c”	3 ± 0.7 b’’’	1 ± 0.1 a’’’’
**C5** ^e^	1.7 ± 0.4 a	0.3 ± 0.1 b’	23 ± 2.2 a”	5 ± 0.3 c’’’	2 ± 0.2 c’’’’
Lim ^a, b, c^	0	0	0	0	0
Lim ^d^	0.6 ± 0.3 b	0.2 ± 0.1 b’	12 ± 1.8 c”	3 ± 0.4 b’’’	1 ± 0.1 a’’’’
Lim ^e^	1.2 ± 0.2 d	0.3 ± 0.1 b’	19 ± 2.1 c”	6 ± 1.3 c’’’	3 ± 0.2 b’’’’

a: 10 µg/mL; b: 1 µg/mL; c: 0.1 µg/mL; d: 0.01 µg/mL; e: 0.001 µg/mL.

## Data Availability

Data are contained within the article and Supplementary Materials.

## Supplementary Materials

The following supporting information can be downloaded at: https://www.mdpi.com/article/10.3390/plants13243527/s1, Figure S1. Percentages of chemical classes in the different samples **C1**–**C5**. Figure S2. Effects of *S. italica* leaf oil on the germination percentage of *R. sativus* seeds of *T. muralis* (positive control).
