# Supplementary material for: Essential Oils Obtained from Sicilian Citrus reticulata Blanco By-Products: Antibacterial and Allelopathic Activity
Source: Plants (Basel). 2024 Dec 17;13(24):3527. doi: 10.3390/plants13243527 (PMC11678590; doi:10.3390/plants13243527)
Supplement: Supplementary file 1 [file plants-13-03527-s001.zip › plants-3339447-supplementary.pdf]

Supplementary Material

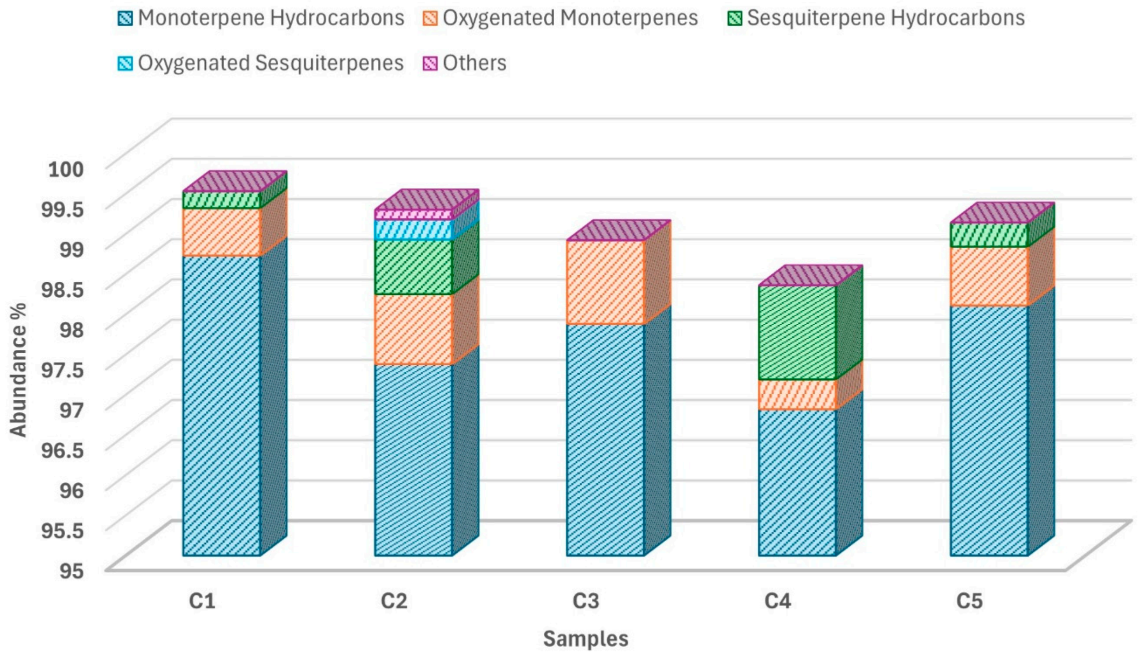

Figure S1. Percentages of chemical classes in the different samples **C1-C5**.

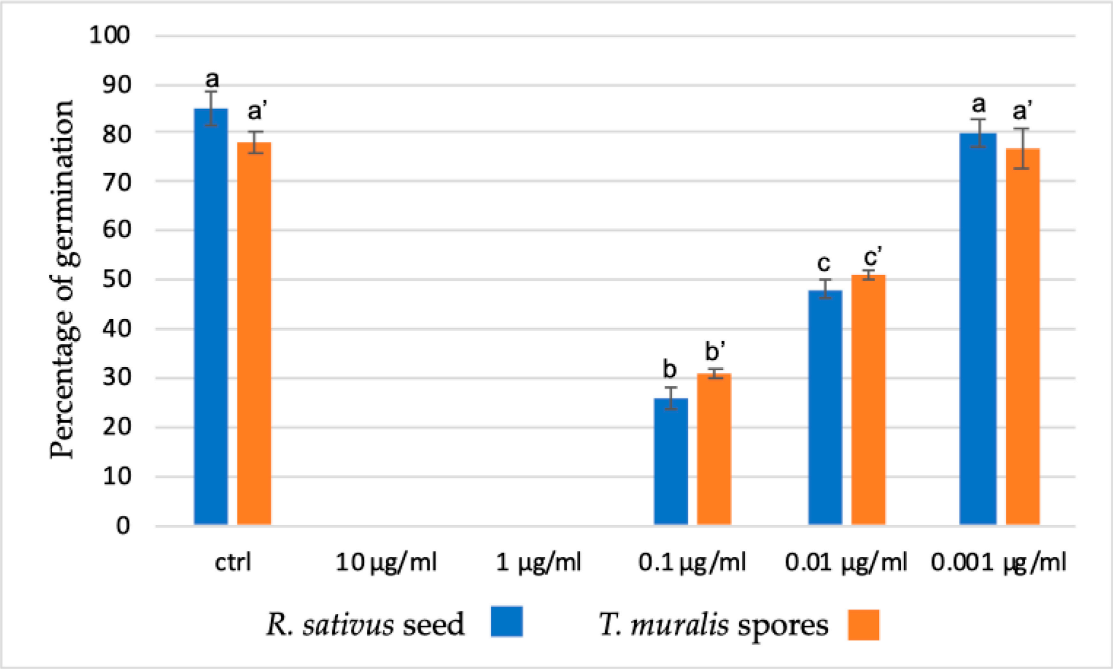

Figure S2. Effect of EO on percentage germination of *R. sativus* seeds of *T. muralis* (Positive control). Data were presented as mean and standard error, and they were analysed with a paired *t*-test. Bars not accompanied by the same letter were significantly different at $p < 0.05$.
